# Prevalence of positive mental health and functioning among adults with sickle cell disease in Ghana

**DOI:** 10.4314/gmj.v54i4.7

**Published:** 2020-12

**Authors:** Richard Appiah, Bempah O Tutu, Mavis E Oman, Peter Ndaa

**Affiliations:** 1 Department of Occupational Therapy, College of Health Sciences, University of Ghana, Accra; 2 Department of Psychology, School of Social Sciences, University of Ghana, Accra

**Keywords:** positive mental health, flourishing, psychological well-being, sickle cell disease, Ghana

## Abstract

**Objectives:**

With increasing survival rates of children with sickle cell disease (SCD) reaching adulthood, there has been a growing interest in the quality of life and mental health functioning of affected individuals. Positive mental health is recognised as a significant dimension of human health that plays an important role in advancing well-being. This study explored the prevalence of positive mental health and functioning among a sample of Ghanaian adults with SCD.

**Methods:**

A quantitative cross-sectional survey design was implemented for data-gathering. A random sample of 62 adult SCD patients (21 to 56 years; mean age of 29 years) receiving treatment at the Sickle Cell Clinic of the Ghana Institute of Clinical Genetics at the Korle-Bu Teaching Hospital completed the Mental Health Continuum-Short Form (MHC-SF). Descriptive statistics and reliability indices were estimated for the MHC-SF. We implemented Keyes's criteria for the assessment and categorisation of levels of mental health to determine the prevalence of positive mental health and functioning.

**Results:**

We found a high level of positive mental health (66% flourishing; 26% moderately mentally healthy; 8% languishing) and functioning, with no significant difference between the genders. A total of 34% of the participants were functioning at suboptimal levels and were at risk of psychopathology.

**Conclusion:**

This study gives the first overview of the prevalence of positive mental health and functioning in a clinical population in Ghana. Although the majority of participants were flourishing, contextually appropriate positive psychological interventions are needed to promote the mental health of SCD patients who are functioning at suboptimal levels, which would, inherently, also buffer against psychopathology.

**Funding:**

Self-funded

## Introduction

Data from global epidemiological surveys show that sickle cell disease (SCD) remains the commonest genetic blood disorder that affects about 20–25 million people^1^,^2^, with a further 400,000 children born annually with the disease^1^. SCD is prevalent among people of the African and Hispanic descents, with approximately 80% of all SCD births occurring in West and Central Africa.^1^,^2^ Regional statistics suggest that about 25% of Africans are carriers of the abnormal hemoglobin gene.^3^ In Nigeria, for instance, about 2 to 3% of the population is affected with SCD and a projected 150,000 children are born each year with the condition.^4^ In Ghana, an estimated 2% of neonates are born with SCD annually.^3^

SCD is a hemoglobinopathy caused by a β-globin gene (βs) mutation at position 6. The mutation results in an abnormal hemoglobin (Hb) with altered physical properties (sickle hemoglobin).^5^,^6^

SCD is categorised into four major genotypes: sickle cell β0 thalassemia (Sβ0 thalassemia), sickle cell β+ thalassemia (Sβ+ thalassemia), sickle haemoglobin C (SC) disease, and homozygous sickle cell (SS) disease.^6^,^7^ Each of these subtypes presents with unique symptoms with varying degree of severity. In particular, the haemoglobin SS, where an individual inherits a sickle S gene from both parents, presents the most severe of clinical symptoms.^8^ The most reported genotypes in Ghana include the ‘SS’, ‘SC’, ‘SD’, and Sβ thalassemia.^9^ SCD differs from sickle cell trait (SCT) or carriers of the sickle cell gene.

People who have SCT do not have the disease but inherit a normal haemoglobin (A) from one parent and an abnormal haemoglobin (SC) from the other parent.

Carriers of the sickle cell gene do not often exhibit signs and symptoms of the disease but are capable of passing the gene to their children. When there is reduced oxygen tension, red blood cells (RBCs) with haemoglobin S (HbS) tend to sickle. This is because HbS becomes less soluble in low oxygen environment and forms tactoids and microfibrils along the long axis of RBC. Sickled RBCs lose their deformability property and thus occlude tiny capillaries. During this process, some of the RBCs are broken down, or lysed. As a result of this occlusion, the distal organs are deprived of oxygen, resulting in complications such as infarction, anaemia, priapism, splenomegaly, and dactylitis.^10^ SCD is associated with poor quality of life, depression, anxiety, and low levels of mental health functioning.^11^,^12^ Although there is limited data on SCD in Ghana at the national level, several studies report on the prevalence of SCD across various health facilities. A retrospective review of the medical records of all SCD patients (aged 13 and above) at the Ghana Institute of Clinical Genetics, Korle-Bu, from January 2013 to December 2014 shows that a total of 5,451 SCD patients accessed healthcare services at the facility, with 20,788 clinic visits.^13^ Another study estimates that about 2% of all newborns are diagnosed with SCD, and at least 25% of the Ghanaian populace carry the sickle cell gene.^3^

Keyes hypothesised a tripartite model of positive mental health that describes three dimensions of mental health, namely, emotional (EWB), psychological (PWB), and social (SWB) well-being.^15^ The EWB describes the presence of positive affect and satisfactions with life, while the PWB defines an individual's intrapersonal and close interpersonal functioning. Keyes conceptualised the SWB to refer to a sense of welfare and happiness and experiences of an individual's well-being in society, as well as satisfaction with their social structure and function.^14^,^15^ Keyes suggested that complete mental health functioning comprised of a combination of all three dimensions: emotional, psychological, and social well-being. For its measurement, a high level of positive mental health, called *flourishing*, requires a combination of a high level of subjective well-being and an optimal level of psychological and social functioning. Likewise, Keyes termed an individual's experience of low levels of positive mental health and psychological and social functioning as *languishing*. Individuals who function between this continuum, that is, neither flourishing nor languishing, are considered to be experiencing moderate mental health.

Keyes' tripartite model, together with empirical evidence from Ryff's^14^ model of psychological well-being, formed the conceptual foundation that directed the development of the 40-item self-administered Mental Health Continuum Long-Form questionnaire.^15^,^21^

A shorter 14-item version, the Mental Health Continuum Short-Form (MHC-SF)^21^, was later constructed to assess the three dimensions of well-being (emotional, psychological, and social well-being) and the categorical diagnosis of positive mental health. According to Keyes^15^,^21^, positive mental health and mental illness exist on two continua – where positive mental health correlates with, but is distinct from, mental illness. In this respect, an individual can present with symptoms of mental illness, such as depressive episode, and simultaneously exhibit a high level of positive mental health (i.e., flourishing). A wealth of research demonstrates that flourishing states are associated with a range of positive outcomes, including good health, higher levels of life expectancy, and satisfaction with life.^17^ The measurement of the prevalence of positive mental health remains a challenging endeavour, partly due to varied conceptualisations and operational definitions of mental well-being. However, in recent efforts, scholars have increasingly utilised the MHCSF to assess positive mental health across various population groups.^19^ There is evidence that suggests that mental health is understood and experienced differently across various cultures and religions.^18^ For instance, researchers found significant difference in the expressions of well-being between collectivist East Asian and individualistic Western samples.^20^

Given the developmental challenges and disease-related complications associated with SCD, survivors require individual-targeted biopsychosocial interventions to be able to lead quality and meaningful lives.^23^ In most cases, SCD patients endure lifelong treatment regimen that can be expensive, complex, and multifaceted, with a high risk for poor mental health functioning, that is, their ability to reach their optimal human potential and lead a meaningful life.^24^ Assessment of positive mental health remains an important approach for evaluating the prognosis and outcomes of chronically ill patients.^25^,^26^

### Prevalence of positive mental health

Scholarly interest in the use of the MHC-SF to assess the prevalence of positive mental health and functioning has increased exponentially in the last decade. Researchers have successfully administered the MHC-SF to adults from South Africa^27^, Poland^28^, Canada^22^, Italy^29^, China^30^, United States^21^ and Australia^31^, and to adolescents from Egypt^32^, India^33^ and South Korea^34^ to explore the prevalence of positive mental health and its correlates.

Prevalence rates vary across the globe depending on the context and the sample involved. For instance, researchers found that only 11.7 % of a sample of South Korean adolescents were flourishing, compared to the 23.5% of Egyptian adolescents, 46.4% of Indian adolescents, and 76.9% of Canadian adolescents and adults.^43^

In Ghana, there are limited studies that explore the concept of positive mental health into its categorical prevalence levels. Largely, the prevalence of mental health has been conceptualised and measured in terms of mental disorders. To our knowledge, only Appiah and colleagues^36^ measured the effect of a community-based positive psychology intervention in promoting the (positive) mental health of a rural adult sample using the MHC-SF. The researchers reported a flourishing rate of 25% at pre-test, 57.5% immediately after the 10-week intervention, and 77.5% three months post-intervention for programme participants. The majority of studies employed proxies in their measurement to assess subjective well-being by measuring individuals' standards of living rather than how they feel about their overall life.^50^ As far as could be established, presently, little is known about the prevalence of positive mental health and functioning in any population group in Ghana. The outcomes of prevalence rates of positive mental health and functioning of a population could serve as an important reference and resource for researchers and practitioners working to develop context-ainterventions to promote the overall mental well-being of the population involved.^35^

## Methods

### Setting and participants

The study was conducted at the Sickle Cell Clinic of the Ghana Institute of Clinical Genetics (GICG) at the Korle-Bu Teaching Hospital. The GICG was established in 1974 by the Ministry of Health and the Managing Trustees of Volta Aluminium Company Limited and serves as a referral health facility for SCD patients across the country. The clinic attends to patients aged 12 and older on every working day of the week with an average daily attendance of 50. Participants (*N* = 62) were Englishspeaking adult SCD patients, aged 18 years and above, of both genders, and were receiving treatment at the GICG for SCD.

### Design and Procedure

A quantitative survey design was implemented. We randomly recruited a total of 62 adults who were receiving in- and out-patient treatment for SCD at the GICG in May and June 2018, by means of a simple random sampling (i.e., ballot) method. After an eligible patient had completed the required medical examination and treatment protocol, the Medical Officer who attended to the patient then introduced the study and the research team to the patient. The research team thereafter provided further information on the study, addressed the patient's concerns, if any, and offered an invitation to the patient to participate. Each consented participant was asked to pick from a box containing pieces of papers with ‘*Yes*’ and ‘*No*’ written on them. The papers were thoroughly mixed after each pick before the next individual was made to pick.

The papers were thoroughly mixed in a container at the beginning of each day of the data collection period, and the process repeated until the desired sample size was attained. All individuals who picked ‘*Yes*’ were contacted and scheduled for interview. Written informed consent was obtained from patients who agreed to participate. Each participant was assured of confidentiality and was informed of their right to withdraw from the study at any point in time without any consequences. Participants were ushered into a pre-arranged office where they selfadministered the questionnaire.

### Measure

#### Mental Health Continuum-Short Form^21^

The MHC-SF consists of 14 items that measures positive mental health in three dimensions: emotional, social, and psychological well-being. Respondents rate how often during the past thirty days they experienced a range of fourteen feelings by choosing one of six options “never”, “once or twice”, “about once a week”, “2 or 3 times a week”, “almost every day”, or “every day”. A categorisation of flourishing (presence of high levels of social, emotional, and psychological well-being) is made when an individual reports experiences as “every day” or “almost every day” for at least seven of the characteristics, where one of them is from the hedonic (i.e., emotional well-being) cluster (i.e., happy, interested in life, or satisfied), and the others from the social and personal/psychological well-being (eudaimonic) clusters. A diagnosis of languishing (absence of positive mental health) is established when a participant reports that they “never” or “once or twice” experienced at least seven of the characteristics, where one of them is from the hedonic (i.e., emotional well-being) cluster and the others from the eudaimonic clusters. A participant who does not fit the criteria for flourishing or languishing is said to be moderately mentally healthy.

A large body of evidence exists that supports the validity of these diagnoses^16^,^44^,^48^ The MHC-SF total score has demonstrated good internal consistency (> 0.80) and discriminant validity. The sub-scales obtained a reliability score of 0.64 for the emotional well-being sub-scale, .57 for the psychological well-being sub-scale, to .71 for the social well-being sub-scale.^37^ Keyes and colleagues found Cronbach alpha of 0.74 for the overall MHC-SF Scale, 0.67 for the PWB subscale, and 0.59 for the Social Well-Being (SWB) subscale in sample of Setswana-speaking adults in the North-West province of South Africa.^28^ The structural validity and psychometric properties of a Twi version of the MHC-SF was recently examined within a sample of rural Ghanaian adults.^51^ The researchers found a high omega coefficient of reliability for the total scale (ω =0.97) and satisfactory reliabilities for the subscales.

### Ethical consideration

The study was approved by the Ethics and Protocol Review Committee of the School of Biomedical and Allied Health Sciences (SBAHS) of the University of Ghana (SBAHS – OT. /10517354/SA/2017-2018). Written informed consent was obtained from each study participant. The processes instituted to ensure confidentiality and anonymity of data were thoroughly explained.

### Data analysis

Descriptive statistics and reliability indices for the MHC-SF were established with the statistical software SPSS 23.0. We applied Keyes' criteria for categorisation of well-being to establish the prevalence of positive mental health and functioning^21^,^37^ using the SPSS.

## Results

### Sociodemographic characteristics of participants

The study involved male and female SCD patients aged from 21 to 56 years. The majority of participants were females, employed, Christians, obtained tertiary level of education. Details of the sociodemographic characteristics of participants are shown in Table 1.

**Table 1:** Sociodemographic characteristics of participants (*N* = 62)

Variable	*n*(%)
**18–30**	36 (58.1)
**31–40**	15 (24.2)
**41–50**	9 (14.5)
**50 and above**	2 (3.2)
**Male**	25 (40.3)
**Female**	37 (59.7)
**Christianity**	56 (90.3)
**Muslim**	6 (9.7)
**Employed**	47 (75.8)
**Unemployed**	15 (24.2)
**No formal education**	1 (1.6)
**Basic**	1 (1.6)
**Second cycle**	20 (32.3)
**Tertiary**	40 (64.5)

### Descriptive statistics and reliability indices for the MHC-SF

Table 2 shows the descriptive statistics and Cronbach's alpha coefficients of the MHC-SF for the sample. We report the mean scores and standard deviations for the scale, which are more or less in line with those reported in the literature. The Cronbach's alpha reliability coefficient for the total MHC-SF was 0.77, which is within the acceptable value of 0.70 and above for reliable instruments.^38^ The mean of the inter-item correlations was .25. According to Clark and Watson's guideline, the interitem correlations of a standardised measure should range between 0.15 – 0.50, with a range of 0.15 – 0.20 for broad constructs and a range of .40 – .50 for narrower constructs.^39^ We found a range of .02 and .68 for the itemtotal correlations for the MHC-SF for this sample. Items 9 to 14 (cluster 3 = eudaimonic; psychological well-being) recorded the highest mean scores (3.90–4.35), whereas Item 8 (Social coherence/interest) presented the lowest mean of 3.15.

**Table 2:** Descriptive Statistics for Sub and Total MHC-SF Scale

	Valid	Mean	Minimum	Maximum	SD
**MHCSF_EWB**	62	10.79	2.00	15.00	3.15
**MHCSF_SWB**	62	16.98	3.00	25.00	4.94
**MHCSF_PWB**	62	24.46	15.00	30.00	3.73
**MHCSF_Total**	62	52.24	30.00	70.00	9.48

Note. MHCSF = Mental Health Continuum-Short Form; EWB = Emotional Well-Being; SWB = Social Well-Being, PWB = Psychological Well-Being

### The prevalence levels of positive mental health

Table 3 shows the prevalence of positive mental health of participants. The results show a high level of positive mental health (flourishing; 66%), a significant level of moderate mental health (26%), and a relatively low level of languishing (8%) among participants. Overall, twothirds of SCD patients have high level of positive mental health, whereas about a third is not functioning optimally. We found no difference *[t=0.111, p>.05]* between the genders in their levels of mental health (see Table 4).

**Table 3:** Prevalence of positive mental health (*N* = 62)

Category	*n*(%)
**Flourishing**	41 (66.1)
**Moderate Mental health**	16 (25.8)
**Languishing**	5 (8.1)

**Table 4:** Gender differences in mental health of participants

Gender	N	Mean	SD	T	p
**Females**	37	62.36	6.54	0.111	.912
**Males**	25	62.60	10.24		

## Discussion

This study set out to explore the prevalence of positive mental health and functioning in a sample of adults with SCD in Ghana. The results show, surprisingly, a high level of positive mental health in the sample, as indicated by a large percentage of participants (i.e., 66%) in the flourishing category, in spite of the high levels of depression^40^ and poor coping methods^41^ previously reported among SCD patients in other settings. A significant percentage of the sample (i.e., 26%) was moderately mentally healthy, with a small number (i.e., 8%) languishing. While the sample for this study differs in many ways from other studies, the results are comparable with previous findings. For instance, the percentage of individuals flourishing in this study is relatively comparable with the 53.1% found among college students in the United States^42^, but lower than the 76.9% reported for a large sample of adolescents and adults in Canada.^22^ In many instances, however, the level of flourishing (i.e., positive mental health) found in the current sample is far higher when compared with the 8% flourishing reported for a sample of South Korean adults^43^, the 20% found among adult South Africans^34^, the 23% found among Egyptian adolescents^32^, the 26% found among a sample of Polish adults^29^, the 42% in a group of South African secondary school children^44^, and the 44% among Chinese adults.^36^ Of note, the participants in the present study are mostly adults, who also present with a medical condition, unlike the participants in the aforementioned studies.

A possible explanation for the high prevalence of positive mental health in our sample could be attributed to the collectivistic cultural and social orientation of the Ghanaian society.^45^ In the Ghanaian communal context, an individual is deeply connected to both the nuclear and the extended family system, where a person falls on relatives and friends for social and financial support. Family and community members may be obligated to assist other individuals in need, particularly when confronted with economic or health crisis.^45^ The availability of support (or the consciousness of its existence) can, in many ways, serve as a source of real or perceived hope for people during difficult times, which, in turn, can help them to build resilience and propel them to function at optimal levels.

Another explanation for the high level of positive mental health found in our sample might be credited to the high level of religious involvement and spirituality^46^ in Ghana. Previous research findings showed that spirituality and religious practices are related to many dimensions of mental health and contribute to advancing the overall mental well-being of individuals and groups.^47^ High levels of spirituality and religious engagements have been associated with positive mental state.^47^

We propose another explanation for the high level of positive mental health and functioning found in this clinical sample, namely, that the general optimism and positive outlook, which are typical of the Ghanaian peoples, have the potential to protect against poor mental health.

Research evidence suggests that most Ghanaians are resilient^52^, optimistic^53^, and happy^54^, even in the midst of life's difficulties. These characteristics, fostered in the Ghanaian collectivistic cultural values^55^ and high religiosity^46^, might have contributed to the high proportion of flourishers in this sample, in spite of their medical presentation.

The findings also showed that, overall, 34% of participants are not functioning at optimal levels. Presence of moderate mental health and languishing are indications of poor mental health, and indeed, a risk factor for psychopathology.^48^ Although the majority of participants reported high level of positive mental health, a third of the sample (i.e., those in the languishing and moderately mental health categories) could benefit from contextually appropriate positive psychology interventions (PPIs) to promote their mental health and functioning. PPIs have been found to protect against psychopathology.^36^

Our results did not demonstrate significant difference between the genders. This finding is consistent with previous research reports that did not establish any significant difference in positive mental health between males and females.^27^–^29^,^31^ Nonetheless, more recently, Guo and colleagues found a slightly better positive mental health among females than males.^35^

### Study limitations

This study may be considered in the light of a few limitations. First, the study was conducted in Accra – the nation's capital and an economically developed part of Ghana. It is admissible that family income might be higher among our participants than in other less developed areas, considering the regional economic disparities in Ghana.^46^ Previous research demonstrates that economic status impacts levels of mental health functioning.^49^ Second, given that the information was self-reported, there is (as it is often the case with most self-reported responses) a risk of socially desirable responses. To minimise this risk, however, we implemented a data collection process that ensured that the self-administered questionnaire was answered anonymously. Lastly, this study recruited a small sample of 62 SCD patients from one healthcare facility. It is recommended that a largescale longitudinal study using questionnaires validated in the languages and cultural contexts of the target population^56^ be carried out with samples representative of the 16 regions of Ghana to establish the level of positive mental health at the national level. National estimates of prevalence of mental health (or illness) have both policy and clinical implications.

## Conclusion

The findings from this study provide insight into the mental health functioning of patients with SCD in Ghana, as measured with the MHC-SF. We found a considerably higher prevalence of positive mental health among our sample, with a third of the sample functioning at suboptimal levels, mentally. It is important that researchers, practitioners, and policymakers explore and take advantage of the contextual and psychosocial factors that promote the emotional, psychological, and social wellbeing to design context-appropriate mental health interventions for individuals in treatment for SCDs, and the Ghanaian people, more generally.
